# Neurologic Toxicity of Immune Checkpoint Inhibitors: A Review of Literature

**DOI:** 10.3389/fphar.2022.774170

**Published:** 2022-02-14

**Authors:** Víctor Albarrán, Jesús Chamorro, Diana Isabel Rosero, Cristina Saavedra, Ainara Soria, Alfredo Carrato, Pablo Gajate

**Affiliations:** Medical Oncology Department, Ramon y Cajal University Hospital, IRYCIS, Madrid, Spain

**Keywords:** immunotherapy, checkpoint inhibitors, neurologic, toxicity, neuropathies

## Abstract

Immune checkpoint inhibitors have entailed a change of paradigm in the management of multiple malignant diseases and are acquiring a key role in an increasing number of clinical sceneries. However, since their mechanism of action is not limited to the tumor microenvironment, their systemic activity may lead to a wide spectrum of immune-related side effects. Although neurological adverse events are much less frequent than gastrointestinal, hepatic, or lung toxicity, with an incidence of <5%, their potential severity and consequent interruptions to cancer treatment make them of particular importance. Despite them mainly implying peripheral neuropathies, immunotherapy has also been associated with an increased risk of encephalitis and paraneoplastic disorders affecting the central nervous system, often appearing in a clinical context where the appropriate diagnosis and early management of neuropsychiatric symptoms can be challenging. Although the pathogenesis of these complications is not fully understood yet, the blockade of tumoral inhibitory signals, and therefore the elicitation of cytotoxic T-cell-mediated response, seems to play a decisive role. The aim of this review was to summarize the current knowledge about the pathogenic mechanisms, clinical manifestations, and therapeutic recommendations regarding the main forms of neurotoxicity related to checkpoint inhibitors.

## 1 Introduction

The irruption of immune checkpoint inhibitors (ICIs) has changed the therapeutic landscape of several tumors and, consequently, the prognosis of our patients. However, many questions still remain unresolved. The adequate diagnoses and correct management of immune-related adverse effects (irAEs) could be a difficult challenge in some situations.

Through the blockade of the interaction between immune checkpoints such as programmed cell death protein-1 (PD-1) or cytotoxic T lymphocyte antigen-4 (CTLA-4) and their corresponding ligands, ICIs are able to suppress the tumoral negative stimulus over cytotoxic T-cell-mediated response, leading to a systemic immunogenic activity. This T-cell activation may cause a wide spectrum of irAEs that can occur in up to 65% of ICI-exposed patients (Boutros et al., 2016), mainly involving the gastrointestinal tract, liver, and endocrine system.

Neurological immune-related adverse events (NirAEs) occur in an estimated 1%–6% of patients treated with ICIs, and they can cause different clinical and pathological disorders affecting both the peripheral and central nervous systems, often requiring treatment discontinuation and sometimes entailing a significant deterioration of patients’ quality of life.

Anatomical determinants such as the blood–brain and blood–nerve barriers, as well as peculiarities in the microglia and other resident cells within the tumor microenvironment, may be at least partially behind the low incidence of neurological immune-related toxicity compared to other organs. However, the precise mechanisms of action of ICIs in the nervous system remain essentially unknown.

Neuromuscular disorders are the most commonly reported forms of NirAEs (5.5%) (Xu et al., 2019). Myositis is the most prevalent neuromuscular syndrome and appears in nearly up to 3% of patients exposed to anti-PD-1/programmed death-ligand 1 (PD-L1) therapy (Wang et al., 2019). On the contrary, central nervous system (CNS) involvement is significantly less frequent (0.5%) and usually appears as encephalitis, vasculitis, meningitis, myelitis, and cranial neuropathies.

Although vasculitis usually has a relatively benign natural course, meningoencephalitis and other forms of CNS toxicity may be grade 3 or 4 adverse events, with a high mortality rate, scarce options of therapeutic management, and a high risk of treatment discontinuation (Sato et al., 2019). In addition, both the development of *de novo* CNS demyelination and the exacerbation of known primary neurological autoimmune diseases, such as demyelinating neuropathies, myasthenia gravis, or multiple sclerosis (MS), have also been reported after exposure to ICIs.

Current therapeutic recommendations for neurotoxicity related to ICIs include stop or discontinuation of immunotherapy and administration of high-dose steroids, considering the administration of intravenous immunoglobulins, plasma exchange therapy, or other immunosuppressant drugs in refractory cases. However, there is a need of further structured research to better understand and optimize the clinical management of NirAEs.

A non-exhaustive systematic literature search was conducted in MEDLINE database. Several articles were obtained for the different syndromes described in the manuscript. However, there is a lack of prospective studies to guide the correct management. Most of the data were recovered from retrospective series and single-institution experience. This is the most important limitation of our manuscript. In this review, we aimed to summarize the current available scientific data about the pathogeny, clinical phenotype, and treatment recommendations regarding the neurological toxicity of ICIs.

## 2 Physiopathology

Due to the increasing incidence of irAEs during the last years, the mechanisms involved in this type of toxicity are an active research field, although many aspects regarding their pathogeny remain poorly understood to date. In the particular case of NirAEs, the relatively low incidence and the difficulty to obtain histological samples, especially from the CNS, have made the comprehension of the pathogenic process behind them especially challenging.

The heterogeneity in clinical presentation, the various affected organs and cells, a wide spectrum on timing, and the extensive variability of the reported histological findings suggest that there are different mechanisms involved, as well as some patient-specific factors that could entail an individual susceptibility to develop this kind of toxicity (Wesley et al., 2021).

The current approved ICIs target PD-1, PD-L1, and CTLA-4. Monoclonal antibodies (Abs) against these molecules suppress immune inhibitory signals upon T cells, allowing T-cell proliferation, tumor recognition, and destruction (Granier et al., 2017). The blockade of both CTLA-4 and PD-1/PD-L1 cell interactions has been replicated in animal models and has been found to facilitate autoimmunity (Klocke et al., 2016; Roberts et al., 2021). Although an antitumor response is the one expected, they are not tissue antigen-specific and therefore are not limited to the tumor microenvironment.

As part of the immune system, there are regulatory T cells (Tregs) that are key players in maintaining immune tolerance by actively suppressing effector T cells. Tregs also express CTLA-4 and PD-1, so they are direct targets of ICIs. This loss of immune regulation, with failure of T-cell tolerance and activation of immune effector cells, may lead to the development of irAEs (Francisco et al., 2010).

Tregs and other immune system cells interact and are highly regulated by cytokines. Pro-inflammatory cytokines lead to destruction, whereas anti-inflammatory cytokines help maintain immune tolerance. ICIs have shown different effects on cytokine levels, and these might be related to the pathophysiology behind irAEs.

Cytokines have also been studied as potential prognostic biomarkers in patients treated with ICIs (Vilariño et al., 2020). The elevated expressions of 11 pro-inflammatory cytokines (G-CSF, GM-CSF, fractalkine, FGF-2, IFNa2, IL12p70, IL1a, IL1B, IL1RA, IL2, and IL13) have been correlated with the development of severe irAEs in a cohort of melanoma patients (Lim et al., 2019). Elevated levels of interleukin 17 (IL17A) have been associated with immune-related neuroendocrine toxicity, suggesting that it can be a diagnostic and therapeutic target, although further studies are needed to validate this hypothesis (Mazzarella et al., 2020).

As previously mentioned, ICIs are not cell-specific. PD-1 and CTLA-4 are expressed in numerous cells and are present in different tissue microenvironments. RNA expressions of PD-1, PD-2, and CTLA-4 were verified over the whole CNS, so it is suggested that these non-hematopoietic cells can be direct targets of ICIs (Caturegli et al., 2016). A patient who received anti-CTLA-4 Abs with evidence of severe hypophysitis, both clinical and histological, showed high levels of pituitary CTLA-4 expression, T-cell infiltration, and immunoglobulin G (IgG)-dependent complement fixation and phagocytosis (Iwama et al., 2014).

Molecular mimicry has been exposed as the underlying mechanism in other autoimmune diseases. For neurological toxicities, cross-reactivity between the tumor antigens and similar epitopes on healthy cells is an important described mechanism behind irAEs. For example, there are some shared epitopes between myelin and melanocytes since both originate from the neural crest, and a common mutation in melanoma is related to the normal *N*-methyl-d-aspartic acid (NMDA) receptor. The first mimicry is associated with peripheral nerve disease and the second one with encephalitis (Wei et al., 2011; Schneiderbauer et al., 2017; Dalakas, 2018). Identical CD8^+^ clonal T cells have been found in the skeletal muscle, myocardium, and cancer cells, supporting the hypothesis that the mechanism behind myositis is also cellular mimicry (Touat et al., 2018; Moreira et al., 2019).

Another suggested mechanism for irAE development is epitope spreading. As part of the response to immunotherapy, the release of tumor and non-tumor antigens subsequent to tissue damage might facilitate new immune responses that can trigger autoimmunity against normal self-tissues, leading to the development of irAEs (June et al., 2017; Memarnejadian et al., 2017).

Paraneoplastic neurological syndromes (PNSs) are rare complications of systemic cancers that can affect all parts of the central and/or peripheral nervous system. They are associated with numerous types of Abs and can cause a wide range of clinical affections. PNSs have been found to be worsened or revealed by ICIs (Vogrig et al., 2019a; Manson et al., 2019). This hypothesis is supported by the demonstration of neural Abs in pretreatment blood samples (Vogrig et al., 2019a; Mammen et al., 2019), suggesting that patients with preexisting Abs are at an increased risk of developing irAEs (Vogrig et al., 2020a).

ICIs have also been reported to exacerbate previously known autoimmune diseases such as MS and myasthenia gravis. Furthermore, some polymorphisms and alterations in immune checkpoint proteins are involved in autoimmune diseases (Gerdes et al., 2016). However, most of the available evidence relies on case reports (Gerdes et al., 2016; Lau et al., 2016; Zhu and Li 2016), and the recurrent exclusion of patients with autoimmune diseases from clinical trials is an obstacle for standardized research.

**FIGURE 1:**
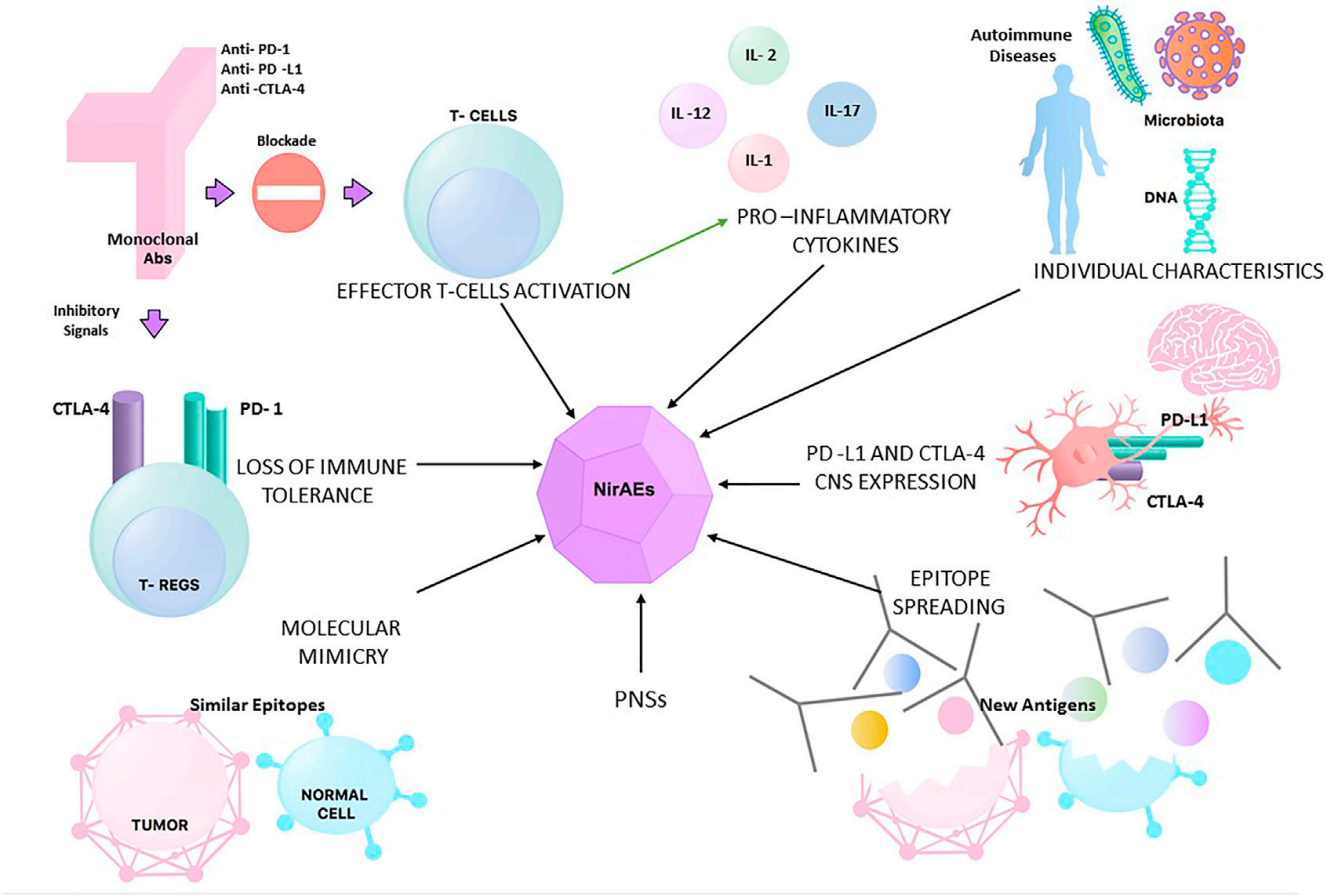
Different hypotheses for the physiopathology of neurological immune-related adverse events (NirAEs). *PNSs*, paraneoplastic syndromes.

Individual characteristics have also been studied as risk factors for the development of irAEs. In terms of genetic features, CTLA-4 polymorphisms have been linked to autoimmune diseases (Lühder et al., 1998), but other studies have failed to find an association between genetic aspects, such as the human leukocyte antigen (HLA) status, and the risk of irAEs (Wolchok et al., 2010). An additional patient characteristic that has been assessed is the gastrointestinal flora, which has been associated with altering the efficacy and toxicity of immunotherapy by modulating the host systemic immune response (Inamura 2020). Figure 1 summarizes the different hypotheses behind NirAEs.

## 3 Peripheral Neuromuscular Syndromes

### 3.1 Myositis

Immune-related myositis constitutes one of the most frequent NirAEs induced by anti-PD-1/anti-PD-L1 Abs. In spite of its prevalence, most of the collected evidence proceeds from small case series or case reports. Moreover, these reports have generally been limited to single malignancies, mainly melanoma patients. To overcome these difficulties, some meta-analyses and systemic reviews have tried to sum up the evidence and bring some light to this rare condition (Cappelli et al., 2017). The importance of this entity lies in its potential consequences, as it may be severe and cause rhabdomyolysis in striated muscle, including the myocardium.

The clinical manifestations of immune-related myositis (irMyositis) differ greatly from those of idiopathic and paraneoplastic inflammatory myopathies such as dermatomyositis (DM) and polymyositis (PM) (Kao et al., 2017; Moreira et al., 2019). Compared to them, oculomotor and axial muscle involvement are not uncommon. Dyspnea, dysarthria, and dysphonia have also been reported. Nevertheless, the clinical pattern is notably uniform, myalgia being the most common and early symptom, even without creatine kinase (CK) elevation (Moreira et al., 2019). Weakness can involve several muscular districts, the involvement of proximal limbs areas being typical, with symmetrical weakness of the pelvic and scapular waistband. Dropping head and cervical extensor muscle weakness are also frequent. Ptosis can be unilateral and is also a common symptom.

The acknowledgment of these clinical features helps to make a correct differential diagnosis, mainly from myasthenia gravis associated with ICIs and cancer-associated paraneoplastic inflammatory myopathies. PM and DM present with progressive weakness of limb muscles as well, but tend to have a more progressive course, while ICI-induced myositis is characterized by an abrupt onset of symptoms (Moreira et al., 2019). Paraneoplastic inflammatory myopathies progress to maximal severity in a range from 1 to 30 days (Touat et al., 2018; Moreira et al., 2019). No fluctuation of symptoms or fatigability is usually described in irMyositis (Touat et al., 2018; Moreira et al., 2019).

Touat et al. reported a median interval between the administration of ICI and the myositis onset of 25 days (range = 5–87). The interval observed was shorter with the combination of two ICIs (nivolumab plus ipilimumab). In these cases, patients can easily develop irMyositis after the first infusion (Touat et al., 2018).

Although the grade of disability acquired at the worst moment of the disease is generally mild, progressive generalized muscle weakness and respiratory or cardiac failure can be life-threatening (Luo et al., 2018; Psimaras et al., 2019). Once the patient requires mechanical ventilation (10%–20% of cases) (Touat et al., 2018; Moreira et al., 2019; Seki et al., 2019) and is admitted at the intensive care unit, the prognosis turns poor and fatality rates approach 14.9%, reaching 50% when myocarditis is added.

Notwithstanding, in the series of cases of Moreira et al., irMyositis completely resolved in almost 50% of patients, with some remaining sequels in around 16% of them (Moreira et al., 2019). Anquetil et al. (2018) showed a higher mortality in patients with ICI-related myopathies compared to idiopathic autoimmune myopathies (21.2% *vs*. <10%) (Anquetil et al., 2018). Similar mortality rates (12 out 29 patients, 41%) were found by Johansen et al. (2019). Nonetheless, these mortality rates must be explained by some other features uncommon for idiopathic autoimmune myopathies, especially cardiac involvement and cancer-related events, which might be the primary drivers for this outcome.

Overlapping with other syndromes is a remarkable characteristic of irMyositis. It is important to recognize common features between irMyositis and immune-related myasthenia gravis (irMG). There are some seronegative cases resembling myasthenia gravis (MG) without positivity for antibodies targeting acetylcholine receptor (AChR), but showing elevated CK or concurrent manifestations (Moreira et al., 2019). Muscle biopsies can play a key role in this scenario, allowing to distinguish patients with necrotizing myopathy from MG. Myocarditis seems to be more prevalent in irMyositis (25%) than irMG (11%) (Cappelli et al., 2017; Moslehi et al., 2018), and it can outline the prognosis of the patient.

Moreover, irMyositis is frequently accompanied by additional irAEs, such as hepatitis, colitis, pneumonitis, and nephritis. Moreira et al. (2019) found that, in 20 of the 38 (54%) patients, other organ systems were also affected by irAEs: thyroiditis (13% of all cases), hepatitis (13%), nephritis (5%), vitiligo (5%), pneumonitis (5%), hypophysitis (5%), and colitis (5%). Importantly, the other irAEs preceded the neuromuscular manifestation in 65% of the cases, whereas in 30% the presentation of both irAEs was concurrent. Only one patient (5%) had neuromuscular side effects previously (Moreira et al., 2019). Dubey et al. evaluated the real-world frequency of neurological irAEs and found that concomitant non-neurological toxicities were present in 68% of patients who suffered from any neurological immune-related complication (Dubey et al., 2020). In the frame of this thinking, patients with irMyositis should be scrutinized for additional irAEs.

Laboratory tests usually show raised CK levels. CK often precedes the onset of skeletal muscle symptoms. The levels of CK and the severity of symptoms are not directly correlated (Moreira et al., 2019), although some authors have reported that the CK levels tend to be higher in patients with severe rather than mild forms (Seki et al., 2019). They are also higher in those patients who received a combination of ICIs (Kadota et al., 2019). Patients can present elevated transaminase and troponin T levels, both related to muscle breakdown and not necessarily to hepatic or cardiac failure. Troponin I seems to be more specific than troponin T for cardiac involvement (Bilen et al., 2016).

Concerning the autoantibody patterns, irMyositis rarely presents positivity for Abs characteristic of inflammatory myopathies. These autoantibodies have been conventionally classified into two groups. The first of them are myositis-associated autoantibodies (MAAs). These are a common trademark of overlap syndromes involving different autoimmune diseases, especially scleroderma and systemic sclerosis. The main Abs of this first group are those that recognize the Ro/SSa antigen, the DNAPK antigen, and PM-scl. The second group is composed mainly of three kinds of Abs: Abs against histidyl transfer RNA synthetase (Jo-1), which defines patients who suffer “anti-synthetase syndrome”; anti-SRP Abs, which are associated with severe myositis with unfavorable prognosis; and anti Mi-2 autoantibodies, very specific for DM and are normally associated with a good prognosis. Abs targeting melanoma differentiation antigen 5 (MDA5) and transcriptional intermediary factor 1 (TIF1) are also associated with DM (Solimando et al., 2020).

IrMyositis is typically negative for those Abs, with some rare exceptions. For instance, in the series reported by Moreira et al. (2019), only 8 of the 24 patients who were assessed for MAAs were positive: one for anti-TIF1, one for anti-Ro52, two for anti-PL-7, one for anti PL-2, and another one for anti-SRP (Cappelli et al., 2017). Meanwhile, Touat et al. (2018) reported no patients with positive Abs, although very few patients were evaluated. Nevertheless, preexisting autoimmunity may increase the probability of myositis. Some patients recruited in clinical trials with ICIs showed, by banked serum analysis, the presence of autoantibodies preceding the infusion of treatment. Those preexisting autoantibodies were predictive biomarkers to identify patients with increased risk of suffering from irAEs and could therefore be included in baseline screening (Moreira et al., 2019; Solimando et al., 2020).

Electrodiagnostic studies such as electroneuromyography typically show myopathic motor unit potentials (defined as the presence of polyphasic, short-duration, or low-amplitude motor unit action potential with normal or early recruitment) (Psimaras et al., 2019) in patients with positive sharp waves and/or fibrillation potentials, being highly uncommon to observe reduced compound muscle action potentials (CMAPs), more representative of MG.

Radiological findings are diverse, but can help support the diagnosis. Positron emission tomography and computed tomography show contrast-enhanced areas. On the other hand, MRI exhibits hyperintense intramuscular alterations on T2 and T1 inversion images and contrast enhancement in T1 images after gadolinium administration (Sidhu et al., 2017; Moreira et al., 2019; Psimaras et al., 2019).

Muscle biopsies have been examined in an erratic frequency in different series. The selection of the site of muscle biopsy must be scrupulous since histological findings are particularly focal and can lead to false negatives. False negatives due to steroid treatment before the biopsy are also noteworthy (Touat et al., 2018). Skeletal muscle biopsies show unique patterns that do not fit with classical inflammatory myopathies. Necrotizing myopathy changes are frequent, and macrophages and T-cell infiltrates are characteristic. The preponderance of different T cells in the infiltrates (CD4^+^ over CD8^+^) is controversial and has been described in both senses (Suzuki et al., 2017; Moreira et al., 2019). The presence of CD20^+^ cells has also been described (Bilen et al., 2016; Touat et al., 2018; Moreira et al., 2019). When comparing irMyositis with DM, the first is mainly T-cell-mediated, while DM presumably shows complement-mediated microangiopathy.

The specific pathophysiology behind irMyositis remains unclear. The main hypothesis arises from the immune cross-activation between normal skeletal and myocardial muscle cells and tumor cells. Transgenic mice that expressed skeletal muscle neoantigens presented loss of cytotoxic activity against autoantigens due to PD-1 expression on CD8-positive cells. This strengthens the postulate that PD-1/PD-L1 signaling activation maintains self-tolerance to muscle autoantigens (Calbo et al., 2008).

Touat et al. described upregulated MHC-I expression on muscle fibers and inflammatory infiltrates mainly constituted by CD68-positive macrophages expressing PD-L1 and lymphocytes expressing PD-1 (Touat et al., 2018). Knauss et al. provided evidence of the activation of the PD-1 pathway and the presence of dysfunctional T cells in some inflammatory myopathies, such as immune-mediated necrotizing myopathy (IMNM), sporadic inclusion body myositis (sIBM), and also in irMyositis (Knauss et al., 2019).

They emphasized as well the importance of T-cell exhaustion and T-cell senescence in this autoimmune disease. Exhaustion and senescence are concepts that refer to dysfunctional states of T cells, which, as the authors hypothesized, can be conducted by persistent antigen presentation. In that study, they yielded the first evidence that T cells in irMyositis express an exhausted phenotype with high expressions of PD-L1, LAG-3, and TIM-3. This is noteworthy because tumor-infiltrating T cells express a similar pattern. It is also notable that 2 cases with severe irMyositis and coexisting myocarditis showed no signs of exhaustion but T-cell senescence. This could lead to further investigations trying to elucidate the association of symptom severity with T-cell senescence. Nevertheless, further studies are needed to shed light on the pathophysiology of this entity.

The clinical manifestations of myositis and other peripheral ICI-related neuropathies are summarized in Table 1.

**TABLE 1:** Clinical presentation of the main ICI-related peripheral neurological syndromes

	**Myositis** (Kao et al., 2017; Suzuki et al., 2017; Johansen et al., 2019; Kadota et al., 2019; Psimaras et al., 2019; Dubey et al., 2020)	**MG** (Romi et al., 2012; Gonzalez et al., 2017; Makarious et al., 2017; Kao et al., 2018; Takamatsu et al., 2018; Knauss et al., 2019; Farrugia and Goodfellow 2020)	**GBS** (Dalmau et al., 2017a; Dubey et al., 2018; Vogrig et al., 2019c; Wesley and Ferguson 2019)
Incidence (% patients treated with ICIs)	1%	–	0.1%–0.3%
Clinical presentation	Myalgia and axial weakness, dropped head (usually symmetrical)	Fluctuating weakness and fatigability of proximal muscles and ocular and bulbar muscles	Hypoesthesia/hyporreflexia and distal motor deficit
	Frequently diplopia and ptosis	Generalized MG; rapid onset and progression to severe forms	Cranial nerve involvement
	Possible respiratory failure	Frequent overlap with myositis (50%–93%)	Neuropathic pain
	Overlapping with myocarditis (25%)	Myocarditis also possible (11%)	Respiratory involvement exceptionally reported
Laboratory test	Elevated CK, troponin T, and transaminases (troponin I more specific of cardiac involvement)	Frequent overlapping with myositis with elevated CK (50.93%)	Anti-ganglioside antibodies rarely positive (12%)
	Specific myositis antibodies are generally negative	Anti-AChR antibodies detected in 53%–87% cases	Cerebrospinal fluid analysis with elevated proteins and mild lymphocytic pleocytosis
ENMG and imaging	Majority of patients (70%–100%) show a myopathic pattern with positive sharp waves. Less frequent reduced CMAPs (50%)	Repetitive nerve stimulation with single fiber shows a decremental response and increased jitter (50%–97%)	Demyelinating pattern with prolonged F-wave latencies and decreased conduction velocities
	PET and CT show contrast-enhanced areas on post-contrast images. MRI with hyperintense intramuscular alterations on T2 and TI inversion images and contrast capitation in T1		Less frequently shows a mixed or axonal pattern
			MRI can expose contrast enhancement of cranial nerves or spinal roots
Other diagnostic workup	Biopsy: focal infiltrates with necrotic pattern and T-cell infiltration	Edrophonium and icepack tests sometimes positive	–
	CD4^+^ and CD8^+^ in variable proportion. CD68^+^ cells present		
	Rare appearance of CD20^+^ cells		

MG: myasthenia gravis. GBS: Guillain-Barré syndrome. ENMG: electroneuromyogram.

### 3.2 Myasthenia Gravis

MG is an autoimmune disorder produced by the binding of autoantibodies to AChR or postsynaptic molecules located in the neuromuscular junction. The several variants of MG are characterized by muscle weakness as the primary clinical manifestation. Symmetric muscle involvement is more frequent, besides eye involvement, which is usually asymmetric. Ptosis and blurred or double vision are classical initial symptoms. Fatigability, meaning that muscle weakness worsens with repetitive activity, is a typical feature, as well as variability throughout the day, which leads to progressive deterioration after an almost normal muscle strength in the morning (Gilhus 2016).

MG is classified in different groups attending to diverse variables such as disease mechanism, thymic status, genetic characteristics, response to therapy, and muscle group involvement. It is remarkable that around 15% of cases present clinical manifestations restricted to eye muscles (ptosis and diplopia) (Kerty et al., 2014). The presence of Abs occurs in half of patients, and in those seropositive cases, the risk of generalized disease strikingly increases.

ICI-related MG is a recognized neurological irAE that has been generally described in case reports and series of cases. Increasing evidence has pointed to the fact that this entity rarely occurs without concomitant myositis (Psimaras 2018). After reevaluation of the reported cases, where the diagnosis mainly relied on the clinical presentation, it is thought that many cases were misclassified. Hence, patients who raise the suspicion of suffering an irMG should undergo a comprehensive assessment with CK, electromyogram, and, if possible, muscular MRI and biopsy in order to detect an associated myositis and therefore adjust the treatment and follow-up. The concurrent presentation of irMG and irMyositis increases the risk of myasthenic crisis that can lead to ventilation support and assistance in an intensive care unit. Most of the fatal cases reported in literature were a consequence of this combination.

The median age of presentation ranges from 65 to 74 years (Makarious et al., 2017; Suzuki et al., 2017; Takamatsu et al., 2018), and around 50% of patients were men. Most of the cases suffered from this complication in the context of ICIs in monotherapy, with the combination of two ICIs responsible for fewer cases, in contrast to irMyositis (Gonzalez et al., 2017). The time to clinical onset oscillates around 40 days, being shorter in more severe clinical forms. Axial limb weakness was described as the most frequent symptom, but ocular involvement was also recurrent (88%–75%). Pure ocular forms were reported by Kao et al. only in 18% of cases (Kao et al., 2018). Bulbar symptoms such as dysphagia and dysarthria are also present. Dyspnea induced by bulbar failure was described in up to 50% of patients and was even reported in all of the 17 patients in Takamatsu’s series. This leads to a more common rapid progression and myasthenic crisis that can result in ventilation support in up to half of the patients. Consequently, this series reported the highest myasthenia-associated mortality, with 47% of deaths. In other series, the mortality rates ranged from 17% to 30% (Gonzalez et al., 2017; Makarious et al., 2017; Suzuki et al., 2017; Kao et al., 2018; Takamatsu et al., 2018). As was previously mentioned, irMG rarely occurs without concomitant myositis. In this sense, CK is elevated in 41%–93% of cases. Psimaras and Suzuki reported that approximately 50% of cases had a proven concurrent irMyositis (Suzuki et al., 2017; Psimaras 2018).

The Abs classically found in MG are the anti-nicotinic AChR, followed in considerably smaller proportions by Abs against muscle-specific tyrosine kinase (MuSK; 1%–10% of cases) and lipoprotein receptor-related protein 4 (LRP4; 1%–3% of patients) (Guptill et al., 2011; Gilhus 2016; Farrugia and Goodfellow 2020). The other detected Abs are anti-ryanodine receptor, anti-titin Abs, and Kv1.4 Abs. Up to 10%–15% of patients with MG remain seronegative after standard antibody laboratory analysis, and no serum Abs against neuromuscular junction are detected (Romi et al., 2012; Gilhus et al., 2016). Remarkably, in irMG, anti-AChR Abs were positive in 57%–83% of cases and in lower titles than in classic MG. There is one case in the literature where Abs against AChR and muscle-specific kinase (anti-MuSK) were found, but the patient had a preceding MG before nivolumab was administered (Mitsune et al., 2018).

The diagnosis usually consists of the combination of classical signs and symptoms with specific Ab-positive tests. In seronegative cases, other clinical tools can be used, such as the edrophonium test, the ice pack test (both rarely used), and neurophysiological tests. Single-fiber electromyography is the best tool to demonstrate the neuromuscular transmission defect. Decremental response of the CMAP to slow (2–3 Hz) motor repetitive nerve stimulation (RNS) is the classical neurophysiologic finding in this test (Pasnoor et al., 2018). Electromyographic findings have a lower prediction rate than in MG since decremental CMAP after low-frequency repetitive nerve stimulation was only reported in around 50% of tested patients (Guptill et al., 2011; Suzuki et al., 2017; Kao et al., 2018). Some authors hinted that concurrent myositis could be responsible for this fact (Guptill et al., 2011).

A study published in 2019 in patients treated with anti-PD-L1 therapy for thymic malignancy showed that patients with preexisting anti-AchR Abs presented elevated CK levels after the administration of ICIs. This outcome was only described in patients with those preexisting Abs (100% *vs*. 0%, *p* = 0.029). Hence, these autoantibodies might predict a higher risk of ensuing neurotoxicity after the activation of latent autoimmune response toward neural antigens by ICI administration (Mammen et al., 2019).

Regarding treatment, pyridostigmine is usually administered, but rarely solely (3%–9%), and frequently immune modulatory treatment is needed, either with steroid alone (27%–45%) or accompanied with immunoglobulin infusion and/or plasma exchange (50%–63%) (Guptill et al., 2011; Gilhus et al., 2016).

### 3.3 Peripheral Neuropathies

Although peripheral neuropathies are typical of conventional chemotherapy, they have also been described in association with ICIs, being usually less frequent and less severe. Many meta-analyses have reported a lower incidence of peripheral neuropathy. Tian et al. described that all-grade peripheral neuropathy (even 3–5) was lower in patients receiving ICIs when compared with chemotherapy (OR = 0.07, 95% CI = 0.04–0.13, *p* < 0.00001). In addition, all-grade peripheral sensory neuropathy was notably lower as well (OR = 0.07, 95% CI = 0.04–0.12, *p* < 0.00001) (Tian et al., 2020). When the combination of ICIs and conventional chemotherapy was compared with chemotherapy alone, a significant increase in the risk of peripheral neuropathy was only observed in grades 3–5 (OR = 1.76, 95% CI = 1.10–2.82, *p* = 0.02). This difference was not statistically significant regarding sensory peripheral neuropathy. Other neuropathies such as dysgeusia and paresthesia also have a lower incidence when compared to chemotherapy agents.

Another meta-analysis stated an overall incidence of sensory peripheral neuropathy of 1.2% compared with patients receiving chemotherapy, which presented an incidence of 8.6% (Nishijima et al., 2017). Furthermore, ICIs have also been related to more rarely peripheral neuropathies such as plexopathies, enteric neuropathy of the peripheral nervous system, and vasculitis (Psimaras et al., 2019).

### 3.4 Guillain–Barré Syndrome

The eponym Guillain–Barré syndrome (GBS) encompasses a wide range of acute immune-mediated polyneuropathies and constitutes a heterogeneous condition with several variant forms. Most often, GBS presents as an acute, rapidly progressing, and potentially fatal form of monophasic paralyzing polyneuritis (Ropper 1992).

Acute classic GBS starts with fine paresthesia in the toes or fingertips, followed within days by leg weakness that makes walking and climbing stairs difficult. Paresthesia is followed by arm, facial, and oropharyngeal weakness while it extends proximally. Pain is common, either as bilateral sciatica or aching in the large muscles of the upper legs (Willison et al., 2016).

From a pathophysiological perspective, GBS has usually been considered a post-infectious disorder, related in most cases to *Campylobacter jejuni*, but also to cytomegalovirus, Epstein–Barr virus, and *Mycoplasma pneumoniae*, among others. GBS was first classified, attending to electrophysiological and pathological studies, into two groups: acute motor axonal neuropathy (AMAN) and acute inflammatory demyelinating polyneuropathy (AIDP). This classification was then supported by the identification of specific Abs for acute motor axonal neuropathy, directed against neuronal membrane gangliosides (notably GM1 and GD1a) (Ropper 1992).

Concurrently with this dichotomization, most evidence describes AMAN as a mainly humoral disorder, leaving T cells as a less important component in the pathogenesis of the acute motor axonal neuropathy, at least in the progressive phase of nerve injury. Nevertheless, during the induction phase where the immune response is developed, T cells might have a key role, especially the T follicular helper (Tfh) subsets, Tfh2 and Tfh17 (Che et al., 2016).

The major immunological mechanism underlying the pathogenesis that misleads the immune system is the molecular mimicry between microbial and axolemmal surface molecules (Willison and Yuki 2002; Soliven 2014). The molecular mimics are lipooligosaccharides (LOS) of preceding infectious organisms, such as *C. jejuni*. They induce the production of anti-LOS Abs that can bind to structurally identical glycans present on nerve gangliosides. Anti-ganglioside Abs in acute motor axonal neuropathy mainly bind to GM1 and GD1a gangliosides.

On the other hand, acute inflammatory demyelinating polyneuropathy has a less transparent pathophysiology since the immunological cascade involved is not yet understood. Specific Abs in this entity are not characterized, but it is speculated that nerve-specific T cells may play a more important role than in acute motor axonal neuropathy (Willison and Goodyear 2013).

It is noteworthy that, besides the high incidence of *C. jejuni* infections in the general population, very few undergo acute axonal neuropathy. One of the hypotheses theorizes that most individuals who have been exposed to *C. jejuni* maintain immunological tolerance to the self-glycans on LOS and instead direct the immune response against other components of the bacterial surface (Makowska et al., 2008). Notwithstanding, it is yet unclear how some individuals lose their tolerance and set off autoreactive response. It is possible that ICIs could break that tolerance and lead to an acute axonal neuropathy.

The pathogenesis of GBS induced by ICIs is even more unclear. First of all, very few reported cases of induced GBS showed detectable anti-ganglioside Abs. Some hypothesized that T-cell-mediated autoimmunity against melanoma cell antigens may also affect myelin antigens on the Schwann cell membrane, as a result of cross-reactivity due to a molecular mimicry since melanocytes and Schwann cells originate from the neural crest and share epitopes for immune responses.

Others have hypothesized that T cells with deficient PD-1 signaling may be preferentially polarized toward effector T-cell differentiation and that the expressions of PD-1 and inducible T-cell co-stimulator may determine the immunological status of circulating memory Tfh cells in patients with GBS (Che et al., 2016; Schneiderbauer et al., 2017).

Despite the attempts to comprehensively characterize the patients who develop GBS after the use of ICI, this entity continues to be poorly recognized due to the limited evidence on this issue, its low incidence, and the lack of profound safety reports in randomized clinical trials. Hence, most of the available evidence comes from case reports and small series of cases, likewise other neurological immune-related adverse events.

GBS is a rare complication within the irAEs, estimated to happen in around 0.1%–0.3% of patients treated with ICIs (Wilgenhof and Neyns 2011; Xu et al., 2019; Fan et al., 2021). There are epidemiological discrepancies between some studies, mainly due to case verification. Hence, we can find a meta-analysis where this peripheral neuropathy had a higher incidence (up to 3% for anti-PD-L1 agents and 7% for anti-PD-1) (Man et al., 2018). Furthermore, there is a systemic review of 86 patients treated with ipilimumab or pembrolizumab where 20 patients (23%) presented demyelinating polyradiculoneuropathy (Johansen et al., 2019).

Fan et al. examined events secondary to ICI treatments in real-world patients based on the Food and Drug Administration Adverse Reporting System (FAERS). Among the 76,514 reports with ICIs as suspect drugs, a total of 149 (0.19%) cases of GBS were screened. The majority of patients were >45 years old (63.1%), men, and, on average, 64 years old. The most common malignancies were skin tumors (41.6%), thorax tumors (24.8%), and genitourinary tumors (12.1%).

The clinical development trended toward hospitalization (61.7%), and 22.8% of cases concluded with death. Monotherapy treatments, either with nivolumab, pembrolizumab, atezolizumab, or durvalumab, entailed 59% of cases, while the combination of nivolumab–ipilimumab signified 37% of the reported cases. These findings suggest a higher incidence of neurological irAEs in patients receiving CTLA-4 plus PD1/PD-L1. The median time of onset was 38 days (range = 0–628). It is remarkable that GBS could take place after the first infusion (Xu et al., 2019).

The most common clinical presentations were sensory motor symptoms involving cranial nerve with bulbar symptoms and dyspnea. Cerebrospinal fluid (CSF) analysis usually showed albuminocytological dissociation associated with a mild lymphocytic pleocytosis (Wilgenhof and Neyns 2011; Psimaras 2018).

In a recent series of 4 cases and a review of 32 previous reports of GBS-like polyradiculoneuropathy induced by ICIs (Okada et al., 2021), the most common clinical manifestation was symmetrical limb weakness (94%), with facial weakness and bulbar involvement observed in 3 and 7 patients, respectively, and severe dysphagia requiring nasogastric tubes in 4 patients. Nerve conduction studies mainly showed demyelination (61%) and axonal (27%) patterns. CSF study mainly showed elevation of protein levels (97%), with lymphocytic pleocytosis in 13 patients (36%). Anti-ganglioside antibodies were positive in only 2 of the 17 patients evaluated (12%).

## 4 Central Neuropathies

### 4.1 Encephalitis

During the last years, a significant number of Ab-associated syndromes affecting the CNS have been discovered. Some of them have been defined as PNSs because of an intimate association with the debut or progression of a malignant disease, while others present as autoimmune encephalopathies (AEs) with a less frequent pathogenic link with a tumor, in which environmental or host-related factors (e.g., infections and HLA) may play a relevant role in unleashing an immune-related response. Despite the recent advances in their clinical characterization, there are still many cases of seronegative encephalitis in which it is not possible to detect a specific onconeural Ab, being highly likely that unknown Abs and other pathogenic mechanisms remain to be properly identified.

PNS are rare immune-related complications of a systemic cancer, typically lymphoma, breast, ovary, or lung cancer, with a global incidence of 0.89/100,000 person-years (Dubey et al., 2018). In PNS, there are tumor-associated intracellular proteins (Yo, Hu, Ma2, Ri, CV2, and SOX-1) that are usually expressed by neurons, leading their antigenic presentation to T cells in order to generate a strong immune response, not only against the tumor but also against CNS cells, where the target antigens are commonly present.

Anti-Hu and anti-Yo are the most frequent PNS-associated Abs (Dalmau et al., 2017a). This simultaneous response explains that tumor growth is usually controlled at the time of neurological symptoms onset, being not surprising that PNS is often the clue to initiate the cancer screening that reveals the presence of an underlying malignancy.

Since there is a lapse of time from the start of an immune response to the damage of a significant number of neurons, the clinical onset of PNS is usually subacute, commonly mimicking neurodegenerative conditions such as degenerative dementias (Wesley and Ferguson, 2019), motor neuron syndromes (Vogrig et al., 2019b), sporadic ataxias, and atypical parkinsonisms (Simard et al., 2020), although rare cases of a hyperacute “stroke-like” onset have also been reported (Vogrig et al., 2019c).

Globally, the most prevalent clinical syndromes are limbic encephalitis (LE) and paraneoplastic cerebellar degeneration (PCD). LE is defined by the subacute development of seizures, confusion, loss of short-term memory, and psychiatric symptoms that appear as a result of limbic system involvement, although patients can also develop mood or sleep disorders, hallucinations, or depression, which may be misdiagnosed as psychiatric illness (Kayser et al., 2010). PCD is characterized by a cerebellar dysfunction that includes limbs and trunk ataxia, dysarthria, nystagmus, and gait imbalance (Shams’ili et al., 2003).

AEs are defined as subacute syndromes consisting of memory deficits, altered mental status, and psychiatric symptoms. Seizures are a common manifestation of AEs and are characteristically resistant to anti-seizure treatments. Some patients may show prominent psychiatric features that typically have a rapid progression, scarce response to antipsychotic drugs (including neuroleptic malignant syndrome), and association with other neurological findings (Muñiz-Castrillo et al., 2020).

They are typically associated with teratomas, thymomas, and small-cell lung cancer, with a global incidence quite similar to that of PNS (0.8/100,000 person-years), but a three times higher prevalence due to their milder clinical course (Vogrig et al., 2020b). Most AEs are related to Ab-targeting proteins that are exposed to the neuronal surface, the most frequent being anti-NMDAR (*N*-methyl-d-aspartate) and anti-LGI1 in adults, as well as anti-NMDA and anti-MOG (myelin oligodendrocyte glycoprotein) in children (Dalmau et al., 2017b). Differently from PNS, these autoantibodies seem to have a direct pathogenic role in AEs, which may explain why AEs response to steroids and immunotherapy, aimed to remove Abs or deplete Ab-producing B cells, is usually satisfactory.

An increasing number of case series and reports have established a firm association between checkpoint inhibitors and immune-related encephalitis that affect 0.1%–0.2% of patients treated with ICIs, particularly when they are administered in combination (Touat et al., 2017a). PD-1 inhibitors seem to have a lower incidence of NirAEs than PD-L1 inhibitors, although given that anti-PD-L1 are less frequently used, the question of whether anti-PD-1 is associated with a significantly higher rate of neurological adverse events remains to be properly answered (Johnson et al., 2019a). In a series of 47 cases of ICI-related encephalitis, the median time between treatment initiation and the onset of symptoms was 65 days.

A total of 19 out of 44 (43%) MRI scans performed revealed findings suggestive of encephalitis, usually hyperintense signals of the medial temporal lobes or the cerebellum in T2-weighted or fluid-attenuated inversion recovery (FLAIR) images, corresponding to zones of inflammatory infiltrates and epileptogenic activity (Stuby et al., 2020). Electroencephalogram (EEG) showed epileptiform activity in around 35% of immune-related encephalitis cases, although there is no specific EEG pattern.

The presence of oligoclonal bands, pleocytosis, and hyperproteinorrachia in the CSF are unspecific inflammatory alterations that can typically be found in patients with immune-mediated encephalitis (Graus et al., 2016). High levels of adenosine deaminase (ADA) have also been reported as a useful clue for the diagnosis of ICI-related CNS toxicity (Fujiwara et al., 2017). The clinical characteristics of encephalitis, in contrast with other forms of ICI-related CNS toxicity, are summarized in Table 2.

**TABLE 2:** Clinical presentation of the main ICI-related central neurological syndromes

	**Encephalitis** (Vogrig et al., 2019a; Dubey et al., 2018; Dalmau et al., 2017a; Wesley and Ferguson, 2019; Vogrig et al., 2019b; Simard et al., 2020; Vogrig et al., 2019c; Kayser et al., 2010; Shams’ili et al., 2003; Muñiz-Castrillo et al., 2020; Vogrig et al., 2020b; Dalmau et al., 2017b; Touat et al., 2017a; Johnson et al., 2019a; Stuby et al., 2020; Graus et al., 2016; Fujiwara et al., 2017; Touat et al., 2017b; Velasco et al., 2021)	**Vasculitis** (Calabrese et al., 1992; Salvarani et al., 2007; Salvarani et al., 2008; Hajj-Ali et al., 2011; Park and Ranganathan, 2011; Giannini et al., 2012; Berlit and Kraemer, 2014; Thom et al., 2016; Daxini et al., 2018)	**Meningitis** (Larkin et al., 2017; Daxini et al., 2018; Fellner et al., 2018; Laserna et al., 2018; Johnson et al., 2019a; Johnson et al., 2019b)	**Myelitis** (Altrocchi, 1963; Lipton and Teasdall, 1973; Christensen et al., 1990; Misra et al., 1996; Transverse Myelitis Conso, 2002; Defresne et al., 2003; de Seze et al., 2005; Pidcock et al., 2007; Cabrera-Gómez et al., 2009; Mader et al., 2010; Hwang et al., 2018; Cuzzubbo et al., 2020; Picca et al., 2021)	**MS** (Pohl et al., 2004; Hauser et al., 2008; Filippi et al., 2016; Gerdes et al., 2016; Garcia et al., 2019; Oliveira et al., 2020)	**CND** (Weerasinghe and Lueck, 2016; Yost et al., 2017; Dubey et al., 2019; Vogrig et al., 2021)
Incidence (% patients treated with ICIs)	0.16%	<0.01%	0.13%	<0.01%	0.03%	–
Clinical course	Psychiatric symptoms, memory deficits, motor neuron syndrome	Headache, altered cognition, focal deficits	Headache, fever, neck stiffness, confusion	Sensory dysfunction, dysautonomia	Sensory alterations, cerebellar dysfunction, fatigue, dysautonomia	Cranial nerve palsy (VII > VIII > II > rest)
Median delay of symptoms	65 days	3 months	9 days	4 months	Variable	3 months
Auto-Abs	Anti-Ma2 (15%)	–	–	Anti-AQP4 (18%)	–	–
	Anti-Hu (8%)					
	Anti-GAD (5%)					
	Anti-NMDAR (3%)					
	Anti-CASPR2 (1.7%)					
CSF	Pleocytosis, high protein levels	Pleocytosis, high protein levels	Pleocytosis, high protein levels	Pleocytosis, high levels of IgG, IL-6 and 14-3-3 protein	Pleocytosis, oligoclonal IgG	Pleocytosis
	Oligoclonal bands	High levels of IL-17				
MRI	T2 and FLAIR hyperintense signals in temporal lobes and/or cerebellum	T2 and FLAIR hyperintense signals	No alterations	T2 signal abnormality co-related to sensory level	T2 and FLAIR hyperintense disperse focal lesions in brain white matter	Cranial nerve enhancement (25%)
		Hemorrhage or ischemic damage		Occasional T1 hypointense lesions		

MS: multiple sclerosis; CND: cranial nerve disorders. CSF: cerebrospinal fluid. MRI: magnetic resonance image.

A review of 60 cases of ICI-related encephalitis, predominantly in patients with melanoma (38.3%) and non-small cell lung cancer (30.0%), showed an association with onconeural autoantibodies in 21 cases (35.0%), anti-Ma2 being the most commonly found (15%), followed by anti-Hu (8.3%), anti-GAD (5.0%), anti-NMDAR (3.3%), anti-CASPR2 (1.7%), and anti-glial nuclear antibody (AGNA) (1.7%) (Touat et al., 2017b).

Although a low number of cases have been reported, a study from the French National Reference Center for Paraneoplastic Neurological Syndromes (Vogrig et al., 2019a) discovered a 112% increase in the detection of anti-Ma2 Abs since the generalization of ICIs in clinical practice, whereas the increase of other onconeural Ab detection (anti-GAD, anti-Hu, anti-Yo, anti-LGI1, anti-NMDA, anti-CASPR2, anti-GABABr, and anti-AMPAr) was significantly lower (30%–50%), suggesting a particularly firm association between ICIs and anti-Ma2 encephalitis.

A systematic review of 82 cases of ICI-associated encephalitis showed that patients with anti-glutamic acid decarboxylase (anti-GAD) or anti-cell surface Abs had a favorable prognosis, whereas patients with other autoantibodies, focal symptoms, and abnormal MRI findings seemed to have poorer outcomes (Velasco et al., 2021).

### 4.2 Central Nervous System Vasculitis

Primary angiitis of the central nervous system (PACNS) is a rare and severe disease defined as an isolated vasculitis of the CNS. Histologically, it is characterized by inflammatory infiltrates that affect the blood vessels supplying the brain parenchyma, spinal cord, and leptomeninges, leading to the thickening of the vessel walls and stenosis that result in poor blood circulation or hemorrhage secondary to vessel rupture (Giannini et al., 2012).

There is growing evidence of the relationship between the use of ICIs and the pathogenesis of medium and large vessel systemic vasculitis. CNS vasculitis is probably an underdiagnosed entity, and its exact frequency as a NirAE is unclear.

Vasculitic entities have also been reported to appear as paraneoplastic syndromes in up to 2%–5% of cases, concurrent with malignancy diagnoses or progression, so the differential diagnosis of ICI induced from paraneoplastic vasculitis can be particularly challenging. Paraneoplastic vasculitis is more often limited to the skin, with leukocytoclastic vasculitis being the predominant form of presentation in 50%–60% of cases, and it is more frequently associated with hematologic malignancies (myelodysplastic syndromes, leukemia, and lymphomas) (Park and Ranganathan 2011).

A systematic review of 20 case reports of histologically confirmed vasculitis following ICI administration showed that the most commonly reported types of ICI-induced vasculitis were large vessel vasculitis such as giant cell arteritis (GCA) and isolated aortitis, followed by vasculitis of both central and peripheral nervous systems (Daxini et al., 2018). They were associated with several ICIs, including ipilimumab (8/20), pembrolizumab (6/20), nivolumab (5/20), and combination therapy with anti-PDL1 and BRAF inhibitors (1/20), with a median time of 3 months (1.2–6) from the initiation of ICIs to the development of symptoms.

The clinical presentation of PACNS is variable and unspecific, the most common symptoms being headache (60%), altered cognition status (50%), and focal neurological deficits such us ataxia, aphasia, dysarthria, hemiparesis, or visual disturbances, generally with an insidious and slowly progressive course. Diaphoresis and marked constitutional symptoms may be indicative of a systemic vasculitis (Berlit and Kraemer, 2014).

CSF examination is altered in 80%–90% of patients and usually show inflammatory findings such us mild lymphocytic pleocytosis and hyperproteinorrhachia, so a normal CSF should direct the workup toward alternative differential diagnosis (mainly CNS infectious diseases and leptomeningeal dissemination) (Salvarani et al., 2007). Oligoclonal bands are occasionally detected, and an increase of IL17-producing cells in the CSF may be helpful in discriminating PACNS from ischemic disorders (Thom et al., 2016).

MRI study is altered in more than 90% patients with PACNS, although its findings are nonspecific. It is often to find signs of microangiopathy, haemorrhage or ischaemic infarctions and multifocal bilateral T2 or FLAIR sequence abnormalities in the cortical-subcortical area (Hajj-Ali et al., 2011). Tumor-like or abscess-like mass lesions can occasionally be found in MRI study, as well as gadolinium enhancement of leptomeninges, making differential diagnosis challenging (Salvarani et al., 2008).

Additional neuroimaging techniques such as CT angiography, high-resolution contrast-enhanced MRI (HR-MRI), or positron emission tomography (PET) may be useful to detect vascular inflammatory activity in patients with large vessel vasculitis with unclear MRI results. In patients with a clinical suspicion of PACNS but inconclusive image findings, brain biopsy is the gold standard technique for definite diagnosis. Its sensitivity is limited, but can be increased to over 80% by targeting areas previously identified as abnormal in the neuroimaging study (Calabrese et al., 1992).

The blood detection of antinuclear Abs (ANAs), anti-neutrophil cytoplasmatic Abs (ANCAs), anti-phospholipid Abs, rheumatoid factor, or cryoglobulins may be informative of an underlying systemic vasculitis or rheumatological disorder, especially Behçet’s disease, Wegener’s granulomatosis, and Churg–Strauss syndrome, where CNS involvement is relatively common (Berlit and Kraemer 2014).

### 4.3 Aseptic Meningitis

Aseptic meningitis is defined by the subacute onset of headache, neck stiffness, and consciousness level alterations, sometimes accompanied by photophobia and fever, and should be differentiated from infectious meningitis and meningeal carcinomatosis.

According to data obtained from clinical trials, less than 0.1% of patients treated with ICIs develop immune-related meningitis, being more often in patients who received anti-CTLA4 or a combination of checkpoint inhibitors, and coexisting with other irAEs in up to 36% of cases (Daxini et al., 2018). However, cases of ICI-related meningitis are poorly described in the medical literature and are probably underdiagnosed. Several cases of meningitis overlapping with encephalitis (meningoencephalitis) have been reported, implying a poorer prognosis and a shorter median time to death compared to immune-related encephalitis alone (Larkin et al., 2017; Laserna et al., 2018; Johnson et al., 2019a; Johnson et al., 2019b).

In a recent series of 7 cases of aseptic meningitis related to ICIs, diagnosis was defined by a clinical syndrome compatible with meningitis associated with a normal brain imaging study (with no signs of myelitis or encephalitis) and >8 lymphocytes/mm^3^ and/or a protein level >0.45 g/L in the CSF, without detection of tumor cells or pathogenic microorganisms (Fellner et al., 2018). The median delay of clinical onset of meningitis was 9 days from the first dose of immunotherapy.

### 4.4 Transverse Myelitis

Transverse myelopathy (TM) is a heterogeneous syndrome with acute or subacute onset characterized by neurological bilateral deficits manifesting as weakness, sensory loss, or autonomic dysfunction as a result of spinal cord inflammation, generally due to infectious or systemic autoimmune diseases.

The most common infectious agents are syphilis, Lyme disease, HIV, HTL-1, *Mycoplasma*, and herpes virus, whereas typical systemic diseases associated with TM are sarcoidosis, lupus erythematosus, Sjögren’s syndrome, Behçet’s disease, and other connective tissue syndromes. However, a significant percentage of cases are idiopathic (Cuzzubbo et al., 2020).

TM can be the first manifestation of MS and neuromyelitis optica (NMO), in which TM is combined with optic neuritis. NMO IgG, also known as anti-aquaporin Ab, is present in >95% of patients with TM as a debut of NMO and may help to differentiate this entity from MS and other closely related neurological syndromes (Transverse Myelitis Conso 2002).

Early clinical presentation of TM generally consists of fever (often associated with bacterial or viral infections), lower extremity sensory dysfunction (paresthesia, numbness or weakness, loss of pain, and temperature sensation), and autonomic signs such as urinary retention or constipation, incontinence, and sexual dysfunction (Mader et al., 2010). These symptoms usually worsen progressively, with a majority of patients reaching their maximum deficit within 7 days (Altrocchi 1963). A third of patients with TM suffer spinal shock, defined as flaccid paralysis with areflexia and loss of cord function below a discrete level (Lipton and Teasdall 1973).

The prognosis of transverse myelitis is variable, with one-third of patients experiencing spontaneous and full recovery, one-third with a mild improvement, and the remaining ones with a poor outcome that often implies a complete inability to walk and, in the case of upper cervical or brain stem involvement, possibly death due to respiratory failure (Altrocchi 1963). A hyperacute onset (time to maximal deficit <24 h) (Christensen et al., 1990), a higher sensory and anatomical level of the spinal lesion (Defresne et al., 2003), a greater longitudinal extent of spinal cord involvement (Pidcock et al., 2007), and presentation with spinal shock (Misra et al., 1996) have been defined as predictive factors of a poor outcome.

The diagnosis of transverse myelitis requires ruling out a structural abnormality suggestive of a compressive etiology of spinal cord deficit by a gadolinium-enhanced MRI. In patients with TM, MRI study frequently shows a T2 signal abnormality corresponding to the clinical sensory level, which is more often located on the cervical cord (44%) and thoracic cord (37%) (de Seze et al., 2005). MRI signal abnormality is multifocal in 5% of patients. In some cases, hypointense lesions can be observed on T1-weighted images, indicating tissue loss or persistent axonal damage.

The CSF study of patients with TM usually reveals inflammatory signs such us pleocytosis and elevated levels of IgG. Other usual findings are high levels of the inflammatory cytokine IL-6 and 14-3-3 protein, which is thought to be a marker of neuronal injury (Cabrera-Gómez et al., 2009).

Similar to what happens in other CNS inflammatory diseases, myelitis may be unleashed by the initiation of a treatment with ICIs, although it seems to be extremely rare, and only around 20 cases have been reported to date. A recent retrospective research in the database of the French Pharmacovigilance Agency and the database of the OncoNeuroTox network (2011–2020) (Picca et al., 2021) identified 7 patients with ICI-related myelitis (3 after treatment with pembrolizumab, 3 after nivolumab, and 1 after nivolumab–ipilimumab combination, predominantly for advanced non-small cell lung cancer).

Patients had received thoracic radiotherapy in almost half of the cases reported, so it has been suggested that local radiation might represent a predisposing factor to the development of myelitis by potentiating the immune response elicited by ICIs (Hwang et al., 2018), although prospective studies are needed to validate this hypothesis.

The clinical presentation is typical of acute transverse myelitis, including paraparesis (100%), sphincter dysfunction (86%), tactile or thermic sensory deficits (71%), and proprioceptive ataxia (43%), with a median number of 7 cycles (around 4 months) from the initiation of immunotherapy to the onset of symptoms. CSF study typically show pleocytosis (67%) and hyperproteinorrhachia (83%).

MRI study showed T2 hypersignals extending for 3 or more metameres in most of the cases, usually with inflammatory changes that affected the brain parenchyma, the leptomeninges, and caudal nerve roots. This suggests that CNS involvement may be higher than in immunotherapy non-related myelopathies.

### 4.5 Multiple Sclerosis-Like and Demyelinating Syndromes

MS is a chronic inflammatory disease of the CNS characterized by recurrent immune attacks to the myelinated neuronal axons, initially causing episodes of reversible neurological deficits that are often followed by progressive neurological deterioration over time. Four major categories have been established in classical MS (relapsing–remitting, secondary progressive, primary progressive, and progressive–relapsing), although its course in clinical practice is highly variable and unpredictable.

The initial symptoms of MS patients are often sensory alterations, essentially paresthesias or dysesthesias, as well as cerebellar dysfunction (ataxia, vertigo, and diplopia) and dysautonomia, mainly constipation and urinary disturbances (Hauser et al., 2008). Fatigue, loss of vision due to optic neuritis, trigeminal neuralgia, and other forms of neuropathic pain are also common manifestations.

MS diagnosis is based on at least two different episodes in the disease course (time dissemination), evidence of chronic inflammation of the CNS by CSF analysis (pleocytosis and oligoclonal IgG) (Pohl et al., 2004) and at least two different plaques or scars in the white matter of the CNS detected by neuroimaging study (space dissemination). MRI findings are based on the presence of T2 and FLAIR hyperintense disperse focal lesions in the brain white matter (Filippi et al., 2016).

A systematic review of 23 patients with NirAEs presenting with CNS demyelination included 5 cases of MS with two different patterns: three of them with a previously diagnosed MS who experienced a relapse during treatment with ICIs and two with previous radiological isolated syndrome (RIS) (suggestive demyelinating lesions without clinical symptoms of the disease) who developed MS clinical manifestations after ICI initiation (Oliveira et al., 2020).

Except for one patient with an atypical clinical course due to encephalopathic symptoms, the remaining cases had clinical and imaging characteristics that were typical of classical MS, with no pattern suggestive of a different pathogenic mechanism due to immunotherapy. Time to symptom onset from ICI initiation was quite variable, with a median of 1 cycle in the case of MS relapse and a median of 9 cycles in the case of evolution from RIS to clinical MS.

In accordance with previous data, a report of 14 patients with a previous diagnosis of MS who were treated with ICIs noted 2 deaths due to disease worsening and relapse (Garcia et al., 2019), and another case report described a radiologically isolated syndrome converted to definite MS after immunotherapy (Gerdes et al., 2016).

### 4.6 Cranial Neuropathies

A few case reports and small case series have reported cranial nerve disorders as possible complications of treatment with ICIs (Cn-ICIs). Most of the reported cases developed along a concomitant meningoencephalitis (Dubey et al., 2019) or GBS (Yost et al., 2017), and there is limited information about the clinical course of isolated ICI-related cranial neuropathies.

A retrospective cohort study and systemic review of the literature identified a total of 39 patients with Cn-ICIs, facial (VII) being the most frequently nerve affected (33%), with bilateral facial palsy in 38% of patients. Vestibulocochlear (VIII) was the second nerve in order of frequency (21%), usually presenting with hearing loss, vertigo, tinnitus, and balance disorders (Vogrig et al., 2021).

They were followed by optic nerve (II) affectation (18%), usually manifesting as a painless reduction of visual capacity, in contrast with idiopathic demyelinating optic neuritis that is characterized by loss of vision usually accompanied by eye pain (Weerasinghe and Lueck 2016). This suggests that some clinical characteristics of Cn-ICIs differ from the usual manifestations of their classical inflammatory counterparts.

Cranial nerve disorders appeared a median 3 months after the initiation of ICIs. CSF analysis showed an inflammatory pattern in 56% of the patients (mainly pleocytosis and occasionally mild hyperproteinorrhachia). MRI study found enhancement of the affected nerve in 25% of the patients, being normal or showing unspecific findings in the rest of the cases.

## 5 Treatment and Management Recommendations

The management of irAEs is based on two main aspects: the discontinuation of the ICI, temporarily or permanently, and the administration of immunomodulatory treatments.

In the case of neurological toxicities, in all but mild (grade 1) symptoms, the medication should be withheld, even if the etiology of the symptoms is still not clear. Upon evolution, for moderate (grade 2) symptoms, the reintroduction of ICI can be considered for mild forms of presentation, such as peripheral neuropathies or aseptic meningitis if the symptoms resolve to grade 0, whereas for severe (grades 3 and 4) symptoms or in the case of MG, GBS, or transverse myelitis, permanent treatment discontinuation is recommended. Although further research is required, if a patient develops immune-related toxicity after monotherapy with anti-CTLA4, it is generally safe to consider a treatment with anti-PD1/PDL1 Abs (Menzies et al., 2017). It is also important to balance the risk of resuming the treatment for grade 1 and 2 irAEs against the risk of an uncontrolled neoplastic disease (Spain et al., 2019).

Similar to other irAEs, steroids are the main and first-line treatment for neurological toxicities. For grade 2 (moderate symptoms), prednisolone 0.5–1 mg/kg should be considered. In the case of significant neurological toxicity (grades 3 and 4), high-dose steroid therapy with oral prednisolone (1–2 mg/kg) or an intravenous equivalent should be used.

If no improvement is appreciated after an initial dose, steroids can be increased up to 2 mg kg^−1^ day^−1^. Once improvement is noted, conversion from intravenous to oral steroids can be considered with a prolonged taper for 4–8 weeks approximately, adjusted by clinical evolution. Steroid-related adverse events such as gastritis, osteoporosis, and opportunistic infections should be contemplated, especially for long treatments (>4 weeks). The addition of proton pump inhibitors, vitamin D with calcium supplementation, and *Pneumocystis jirovecii* prophylaxis should be considered when indicated.

In the case of MG or GBS, if there is no response to steroids, intravenous immunoglobulin or plasmapheresis may be considered. For MG, other immunosuppressants such as azathioprine, cyclosporine, and mycophenolate have been used. Besides these treatments, the use of other immunomodulatory agents such as rituximab, cyclophosphamide, and tocilizumab has been reported for many NirAE presentations, although these immunosuppressive treatments have not been evaluated in a large number of patients.

As a general aspect, for the diagnosis and treatment of these types of irAEs, prompt consultation with a neurologist is advised, especially for grade 2 or higher NirAEs.

The treatment recommendations exposed in this article, and in general the ones followed in a clinical basis, were based on the practice guidelines for the management of immunotherapy-related toxicities from the European Society of Medical Oncology (ESMO) (Haanen et al., 2017), the National Comprehensive Cancer Network (NCCN) (Thompson et al., 2020), the Society for Immunotherapy of cancer (SITC) (Puzanov et al., 2017), and the American Society of Clinical Oncology (ASCO) (Brahmer et al., 2018). Table 3 summarizes general treatment recommendations and Table 4 summarizes some treatment recommendations regarding specific NirAEs.

**TABLE 3:** Management of suspected neurological immune-related adverse effects (irAEs)

**Grade/CTCAE**		**Management**
Grade 1	Mild symptoms	Consider to withhold ICI
	No interference with function	Close monitoring for any progression
	Symptoms not concerning to patient	If irAEs worsen or do not improve, consider permanent discontinuation
Grade 2	Moderate symptoms	Withhold ICI
	Cranial nerve involvement. Some interference with ADL. Symptoms concerning to patient	If irAEs worsen or do not improve (going to grade 1), consider permanent discontinuation
		Start 0.5–1.0 mg kg^−1^ day^−1^ prednisolone equivalents PO or IV; if worsening symptoms, 1–2 mg kg^−1^ day^−1^
		*Initial observation reasonable
Grade 3	Severe symptoms	Permanently discontinue ICI
	Limits self-care	Start 1–2 mg kg^−1^ day^−1^ prednisolone equivalents PO or IV
Grade 4	Life-threatening consequences	Permanently discontinue ICI
		Start 2 mg/kg^−1^/day^−1^ prednisolone equivalents PO or IV

CTCAE: Common Terminology Criteria for Adverse Events. ADL: activities of daily living. PO: per oral. IV: intravenous.

**TABLE 4:** Other treatment recommendations for suspected immune-related neurological syndromes

**Suspected syndrome**	**Treatment recommendations**
Central neurological toxicity	
Aseptic meningitis	Consider concurrent empiric antiviral (i.v. acyclovir) and antibacterial therapy
Encephalitis	Consider concurrent empiric antiviral (i.v. acyclovir)
Transverse myelitis	Start 2 mg kg^−1^ day^−1^ (methyl)prednisolone or 1 g/day
	If no improvement or worsening, consider plasmapheresis
Peripheral neurological toxicity	
Guillain–Barré syndrome (GBS)	Consider 1–2 mg kg^−1^ day^−1^ prednisolone equivalents PO or IV
	If no improvement or worsening, plasmapheresis or intravenous immunoglobulin indicated
	• Ventilatory support should be available
	• Steroids not recommended for idiopathic GBS
Myasthenia Gravis	Steroid indicated—dosing according with grading of symptoms
	Pyridostigmine, initial dose of 30 mg
	If no improvement or worsening, consider plasmapheresis or intravenous immunoglobulin, additional immunosuppressants azathioprine, cyclosporine, or mycophenolate
	*Avoid medications that may precipitate cholinergic crisis

Besides the information presented in the major clinical guidelines, other immunomodulators have been proposed. In the case of peripheral nervous irAEs, after plasmapheresis and intravenous immunoglobulin in steroid-resistant cases, the most common third-line treatments are tocilizumab, infliximab, rituximab, mycophenolate, methotrexate, and cyclophosphamide (June et al., 2017; Memarnejadian et al., 2017; Spain et al., 2017). Among these medications, rituximab has shown more effectiveness for MG, and other antibody-mediated NirAEs, due to the depletion of B lymphocytes (Deftereos and Georgonikou 2021).

Although not specifically for immune-related encephalitis, but rather for general AEs, rituximab and cyclophosphamide are treatment options if there is no improvement with steroids, plasmapheresis, and intravenous immunoglobulins. In a retrospective study of 161 patients with AEs, additional rituximab treatment was associated with improvement of functional outcomes, with no life-threatening adverse events reported (Lee et al., 2016). Even though it has serious side effects such as myelosuppression, infertility, and hemorrhagic cystitis, cyclophosphamide is still a widely used medication for AEs, its low cost being an advantage when compared to other immunosuppressants. Other medications suggested for refractory AEs cases are tocilizumab and bortezomib (Shin et al., 2018).

The response to treatment varies widely upon clinical presentations. As an example, in the case of irMyositis, 84% of patients experience favorable clinical outcomes after ICI discontinuation and immunomodulatory treatment, with clinical improvement within days in mild forms and over several months in severe cases (Psimaras et al., 2019). On the other hand, in irMG, 50%–63% of patients require corticosteroids accompanied with immunoglobulin infusion and/or plasma exchange (50%–63%), with a prolonged recovery time (Guptill et al., 2011; Gilhus et al., 2016). In a study including different types of NirAEs, both peripheral and central, symptoms resolved in 26 patients (75%), with a median time to resolution of approximately 1 month (Larkin et al., 2017).

## 6 Conclusion

NirAEs associated with ICIs are rare, but they have relevant complications with a potential long-term disability or death. Although the clinical presentation is diverse and there is a lack of clinical trials to guide the management, early recognition and adequate treatment are important for a clinical recovery. The pathogenesis of these events is not well known. In this manuscript, we reviewed the most important central and peripheral NirAEs. The clinical presentation and the different diagnostic tools are critical for a correct diagnosis. The treatment recommendations were based on ICI discontinuation and administration of immunomodulatory therapies.
